# Insects and Public Health: An Overview

**DOI:** 10.3390/insects14030240

**Published:** 2023-02-27

**Authors:** Simone Belluco, Michela Bertola, Fabrizio Montarsi, Guido Di Martino, Anna Granato, Roberto Stella, Marianna Martinello, Fulvio Bordin, Franco Mutinelli

**Affiliations:** Istituto Zooprofilattico Sperimentale delle Venezie, Viale dell’Università 10, 35020 Legnaro, Italy

**Keywords:** insects, public health, farming, biosecurity, disease, welfare, food, feed

## Abstract

**Simple Summary:**

Traditional and emerging topics bridging insects and public health are described because insects affect human and animal health. Addressing public health professionals, this paper aims to (i) outline and enforce the role of public health authorities in different sectors involving insects, health, food and feed, (ii) improve the rearing, management conditions and animal welfare of insects, and (iii) enhance research activities on different aspects of the insect-public health interface.

**Abstract:**

Insects are, by far, the most common animals on our planet. The ubiquity and plethora of ecological niches occupied by insects, along with the strict and sometimes forced coexistence between insects and humans, make insects a target of public health interest. This article reports the negative aspects historically linked to insects as pests and vectors of diseases, and describes their potential as bioindicators of environmental pollution, and their use as food and feed. Both negative and positive impacts of insects on human and animal health need to be addressed by public health professionals who should aim to strike a balance within the wide range of sometimes conflicting goals in insect management, such as regulating their production, exploiting their potential, protecting their health and limiting their negative impact on animals and humans. This requires increased insect knowledge and strategies to preserve human health and welfare. The aim of this paper is to provide an overview of traditional and emerging topics bridging insects and public health to highlight the need for professionals, to address these topics during their work. The present and future role and activities of public health authorities regarding insects are analyzed.

## 1. Introduction

Insects are, by far, the most common animals on our planet with more than 1.5 million species named [1]. Insects have colonized almost every conceivable habitat and created the biological foundation for all terrestrial ecosystems. They decompose and cycle nutrients, disperse seeds, maintain soil structure and fertility, control populations of other organisms assuming different roles (e.g., predators, parasites, parasitoids, disease agents and vectors), and provide a major food source for other taxa (e.g., amphibians, reptiles, birds, fish, arthropods and other invertebrates, mammals) [2]. In particular, insects have a fundamental role as pollinators. Wild and managed pollinators are closely linked to human well-being through their pivotal role in wild plant reproduction and crop production, affecting the yield of approximately 75% of the world’s most important crop types [3]; therefore, we can state that insect pollinators are responsible for one of every three bites of food we eat [4]. In addition, insects, considered as a food (re)source, appear more sustainable when compared with other sources of animal protein, thus possibly contributing to alleviating the pressure on the environment and the planet in feeding a densely populated world [5].

The ubiquity and the plethora of ecological niches occupied make insects a target of public health interest from various perspectives. Beyond the already mentioned roles for life on earth, their presence can directly affect public health by transmitting diseases to both humans and animals; especially, biting insects play a role as vectors and are pests. Due to climate changes and increasing rates of resistance among arthropods to insecticidal substances, vector-borne diseases are expected to expand and increase their importance in the near future [6]. In addition, insects can infest and contaminate food and feed commodities, destroy cultivated crops and impact livestock causing food losses (pests).

However, insects also have beneficial relationships with humans. Honeybees have been farmed or exploited worldwide for millennia for honey production, and are the only insects classified as livestock. In addition, *Apis mellifera* is well-known as a human food in the eastern hemisphere [7]. Similarly, silkworm has a long history of industrial rearing for silk production, which has been an important source of income for many societies. Moreover, some insects have been used for biological control of insect pests and as biological indicators. More than one thousand species are traditionally consumed as food in several countries worldwide [8] and more recently have attracted the interest of developed countries for their potential as a sustainable food and feed [9]. Lastly, some species are reared and employed in experimental studies and to recycle organic leftovers for food and feed production [10].

**Figure 1:**
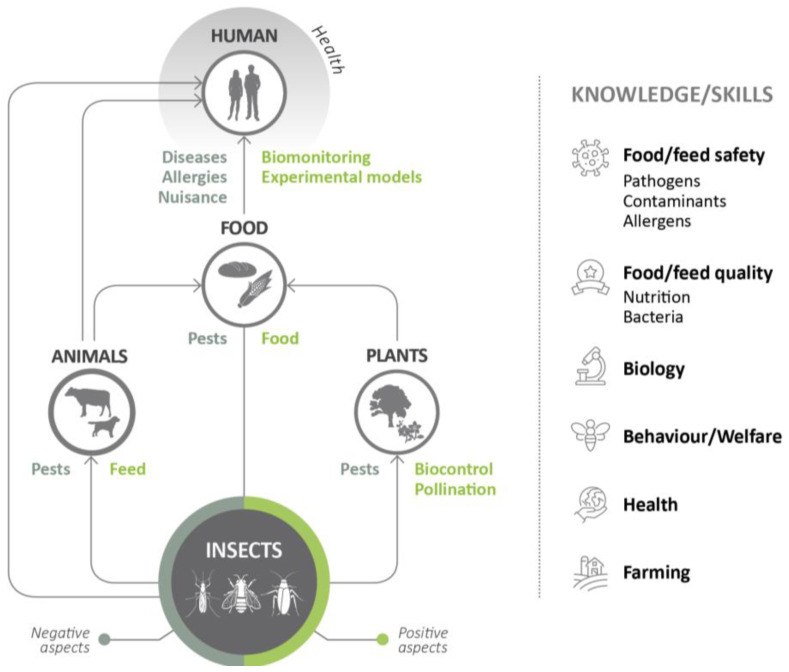
Positive (green) and negative (grey) relationships between insects with respect to contexts with public health implications.

The strict and sometimes forced coexistence between insects and humans (Figure 1) highlights the importance of implementing public health systems to cover all aspects of the human-insect interface as has been done for other animal species. To address the many challenges insects continue to pose, public health authorities have updated knowledge on historical applications, but they have to master the new frontiers in insect research.

The aim of this paper is to provide an overview of traditional and emerging topics linking insects and public health to highlight the need for professionals to include and address these topics during their work. The discussion considers the present and future role and activities of public health authorities with respect to insects, considering the wide range of sometimes conflicting goals, such as regulating their maintenance, exploiting their potential and protecting their health.

## 2. Insects and Diseases

### 2.1. Insects as Vectors of Pathogens

The most well-known factor concerning insects related to public health is their role as vectors of pathogens. Arthropods can transmit several infectious pathogens (e.g., bacteria, parasites, protozoa and viruses) resulting in more than 700,000 deaths per year from vector-borne diseases worldwide [11]. In this context, arthropods can be divided into mechanical vectors and biological vectors. The former, generally non-blood-sucking, such as cockroaches and flies, carry the pathogen mechanically, acquiring the contamination on feces, sewage or other biological fluids and disseminating it in the environment and food by contact. Biological vectors, such as mosquitoes, sand flies, fleas, and ticks, are generally blood-sucking parasites that acquire the pathogen through a blood meal on an infected host. In the vector, the pathogen multiplies and/or completes a phase of its development cycle and is transmitted to several hosts by the vector organism biting them.

Despite efforts in prevention and control methods (diagnosis, treatment, vaccination, prevention and vector control methods) in the last decades, vector-borne diseases are still emerging and they remain amongst the major public health concerns worldwide [12]. In addition, the burden of vector-borne disease is determined by a complex set of demographic, environmental and social factors, among which the increase in human-animal-vector interaction plays one of the main roles, and disproportionately affects the poorest populations, mainly in tropical and subtropical areas [13].

In the present day, vector-borne diseases account for more than 17% of all infectious diseases but, in the near future, this proportion could change due to climate change [14,15]. Furthermore, pandemics, conflicts and other emergencies could lead to increased public health burdens worldwide [11] due to the interruption and/or disruption of services. This has been demonstrated by the COVID-19 pandemic, which has strongly affected malaria services leading to an increase in malaria incidence and mortality rates.

In this context, it is important to identify roles, responsibilities and activities to be implemented, optimizing human and economic resources in designing effective and sustainable entomological surveillance systems. In the case of disease outbreaks involving insects as vectors, public health authorities should be ready to recognize the transmission pathway, establishing target monitoring activities (through species-specific capture methods) to detect responsible insects and control their spread in the surroundings.

For the prevention of vector-borne diseases it is necessary to adopt a One Health approach, known as integrated surveillance, based on entomological surveillance [16,17]. The collection of entomological data provided by entomologists of the public health service is important to assess the risk of spread and circulation of vector-borne diseases, in a certain area, but it is equally crucial to direct any operations to face vectors and evaluate the effectiveness of control methods against vectors. Data provided by these programs are pivotal to timely and effectively support vector control actions to reduce the risk of spreading vector-borne diseases.

Understanding biology and ecology of a vector is extremely important to implement effective strategies to address the process of disease transmission. Research perspectives in this area should be aimed at fully understanding the vector-pathogen-vertebrate host interaction [18]. In this context, experimental infection studies between vectors and pathogenic agents allow the identification of the mechanisms affecting the vector’s ability to acquire, maintain, and transmit the pathogen (i.e., vector competence). These studies require specific structures (insectaries) to rear insects, to generate pathogen-free insects and to conduct experiments requiring high biosafety levels. In laboratory conditions, hematophagous arthropod maintenance is based on female egg production after a blood meal traditionally supplied by both animal and human hosts. However, the expensive and time-consuming host rearing procedures, the accidental transmission of diseases, and the ethical problems concerning animal welfare, direct the research towards the development of cheap, suitable, standardized and effective artificial feeding systems [19].

### 2.2. Insects as Pests

Insects as pests comprise insects able to damage vegetables, animals and humans through different mechanisms of action. First, insect pests can seriously damage forests trees, wood products, cultivated crops and agricultural products by eating leaves or digging burrows in stems, fruit, or roots. This action can lead to contamination (e.g., body parts, exoskeletons, eggs, off-odors) of produce. Furthermore, to the loss of food and feed products must be added economic losses associated with growing, transporting, and storing them. In addition to direct damage to the plant by feeding, insects can indirectly affect plant health by delivering plant pathogens to wounded sites, from where pathogens spread throughout the plant [20]. Secondly, some insect species, such as flies and tabanids, are pests for people and livestock because of their painful and irritating bite, persistent biting behavior, and blood ingestion. Injuries resulting from contact with insects and arachnids are a significant public health concern, and have been increasing in recent years due to the increasing human population expanding into animal-populated territories, as well as changes in animal geographic distribution and pet ownership [21]. Pests associated with livestock production occur at high densities, follow seasonal patterns, and perform disturbing and annoying actions. These can lead to increased stress, loss of feed intake, sleep and/or production, which can impact animal health [22]. Hornets and wasps can severely affect other wild and farmed insects, as well as biodiversity, e.g., *Vespa crabro*, *Vespa orientalis* and *Vespa velutina nigritorax* (yellow legged hornet), the latter recognized as invasive alien species at the EU level [23] due to its predation pressure from July to November [24]. Furthermore, together with honeybees, they can be harmful to humans and animals due to their sting. Particularly aggressive is *V. velutina nigritorax* that can attack humans and animals if the nest is threatened [25,26,27].

Insect pests have significant health and economic impacts and, as with invasive species, also negatively impact ecosystem functions. Public health authorities have set specific standard requirements for the management and control of pests (pest management) and are enrolled in the control their application. Different strategies (i.e., identification and implementation of cultural, mechanical, chemical and biological options) should be sustained by public health authorities to guarantee effective pest management.

Public health authorities and researchers should constantly improve the ability to monitor, mitigate, and manage pest impacts [28]. Research should be addressed to better understanding of the life cycle of these pests to identify possible weaknesses useful to their control.

## 3. Insects as Feed and Food

Population growth and urbanization have increased the global demand for food, especially animal-based protein sources. Insects have a long history of consumption in several countries worldwide [1], whereas in Western countries only the use of honey as a food has a long tradition. 

It has been argued that insects played an essential role in the diet of our ancestors [29] as a complement to animal hunting. In Western countries the Neolithic revolution progressively removed insects from the common diet, whereas in several countries worldwide they have played and continue to play an essential role as a protein source [2]. In addition, edible insects are a promising solution to address global challenges such as climate change, population growth, sustainability, and emerging zoonosis [2].

In recent decades, there has been growing interest toward their introduction in the diet of developed countries, mainly under the pressure to develop a more sustainable diet. This has created a new challenge for public health in the context of food safety. Insects may represent a valuable alternative for meat and fish in satisfying the growing food demand due to their nutritional value and sustainability of production. Besides the interest in insects as food, there is an enormous interest in insects as feed. The traditional production of animal feed, such as fishmeal, soy and grains, needs to be further intensified in terms of resource efficiency and extended using alternative sources. By 2030, over 9 billion people will need to be fed, along with the billions of animals raised annually for food, recreational purposes, and as pets. The use of insects on a large scale as a feed ingredient is technically feasible, and insects as feedstock for aquaculture and poultry feed are likely to become more prevalent within the next decade.

Several microbiological and chemical hazards have been associated with insects. In most cases, these hazards are already known in other food products and require to be managed according to the specificity of the insect supply chain. The main sources of chemical hazardous substances in insects are the production of natural toxins by the insects themselves and the intake of contaminants from farming substrates. Recently, several studies have focused their attention on the presence of mycotoxins [3,4], heavy metals [5], polychlorinated biphenyls (PCBs), dioxins, pesticides [6], persistent organic pollutants, plasticizers, and flame retardants [7]. However, the contamination levels found for pesticides, PCBs, flame retardants, plasticizers and dioxins were relatively low, displaying concentration values similar or lower than those measured in meat, fish and eggs, and not exceeding current legal limits set for similar products [6,7]. In contrast, for heavy metals, data collected by research studies involving different insect species have highlighted that the extent of potential bioaccumulation along the food chain from contaminated soil, plants or water, varies greatly depending on the insect species as well as the investigated heavy metal [8]. It is also important to note that the insect species, the breeding environment and subsequent manipulation during processing and packaging, are additional production stages that can greatly influence the chemical safety of the final product. Regarding toxicity, few studies exist on specific insect components. No substances of concern have been identified in species most commonly used as feed or food [9], but considerations need to be carried out on a species-by-species basis.

As regards microbiological risk, several publication reporting the risk profile of insects as food have been published in recent years [9,10,11]. Recent efforts have tried to understand the whole picture through the application of NGS techniques [12]. However, few studies are available on the risk of presence in raw materials of important pathogens (i.e., salmonella). In addition some new issues must be considered, such as the presence of the *Bacillus cereus* sensu lato group, which requires a distinction between *B. cereus* sensu stricto and *B. thuringiensis* or, even better, between Bacillus toxin producers and those that are not [13]. Compared with mammals and birds, there are no known cases of transmission of diseases or parasitoids to humans, livestock and wildlife from the consumption of insects (with the condition that the insects were handled under the same sanitary conditions as any other food). Insects pose a low risk of transmitting zoonotic diseases such as H1N1 (bird flu) and bovine spongiform encephalopathy [9].

Despite the many advantages of using insects as food due to their content of several bioactive compounds [14], there is risk linked to potential allergic reactions induced by insect consumption. Various insect proteins have been identified as allergens [15]. Food allergies have been described for a number of insects, including to *Tenebrio molitor* [16], the first insect approved in EFSA for human consumption [17]. An allergic reaction linked to the consumption of insects may be caused by the insects themselves (primary sensitization) or by a cross-reaction with another allergen [15]. In this regard, individuals allergic to seafood (e.g., shrimp, crab) are potentially at risk when consuming insects due to potential cross-reactivity with the insects’ tropomyosin and arginine kinase [18]. Current EU legislation [30] does not include insects in the list of allergenic ingredients; however, in the EU, regulations authorizing the use of some insect-based products as food requires producers to add labels with specific warnings for consumers about this risk.

Public health authorities should play a fundamental role to guarantee the sanitary sustainability of insect mass-rearing for food and feed. The introduction of insects and derived products in the human diet is a matter of public health, and being animals, also of veterinary public health. Their path into the food systems requires, first of all, activities in the field of regulatory risk assessment. Risk assessment is required to allow competent national or international authorities to assess their safety. This is the case in the EU and Canada, where a specific regulation for novel foods is in place and requires pre-market authorization. Risk assessment is also required to enforce specific policies guaranteeing public health. The EU, for example, uses a scientific dossier produced by applicants both as the data source for risk assessment and as a basis to define specific food safety criteria [31]. Beyond policymaking, knowledge should be used to allow risk management, and represents a new topic for most practitioners in this field. Clear rules are needed to the benefit of producers and consumers. Information and knowledge are needed for professionals involved in food safety activities, both from a private and public perspective.

It is very important that knowledge about safety of insects as food and feed is spread among public health professionals and becomes part of their safety culture, in particular in those involved in the control of the food chain. Several factors are specific for insects and make them different from commonly eaten animal-based products, such as taxonomical distance, rearing differences, dimension, and poikilothermy. The absence of a real slaughtering phase, the possibility to have small and the relatively simple plants managing the production from farm to final products, represent challenges for public health professionals involved in food and feed controls.

The use of insects as food and feed requires further research activities to assess risks potentially emerging from this supply chain and identifying solution for their management. These should focus primarily on risks from potential zoonosis, pathogens, toxins and heavy metals (through the bio-waste streams). In addition, the role of insects in contributing to human nutrition in providing bio-active compounds should be a research goal.

## 4. Insect as Biological Indicators and for Biomedical Research

### 4.1. Insect as Biological Indicators

Biomonitoring is the scientific evaluation of environmental and human exposure to natural or synthetic pollutants based on the sampling and chemical analysis of living organisms. Insects are excellent indicators of ecosystem health, and have been used as bioindicators for the assessment of pollution both in aquatic [22] and terrestrial ecosystems [23].

Honeybees are an example of insects that act as active samplers and detectors of environmental pollution for many reasons. Honey bees are able to fly up to 10–12 km from their hive, based on the need for food. During foraging activity, their body which is covered with hair, accumulates electric charge due to friction with the air, and traps substances suspended in the air, including pollutants [24,25]. They actively collect pollen, nectar, water, vegetable resins and honeydew, which are stored in the hive. Hive products such as honey, wax, and pollen collected by bees can accumulate contaminants based on their chemical characteristics, and can be analyzed. The honeybees themselves can also be analyzed for biomonitoring studies. As a result of all these characteristics, bees are suitable bioindicators for different types of pollutants, such as heavy metals, polycyclic aromatic hydrocarbons [26] pesticides [23,27,28], radionuclides, brominated flame retardants [32], vehicle-derived ultrafine particulate [33] and microplastics [34].

Biomonitoring programs that assess the presence of pesticides or other harmful substances in honeybees and their products could help in understanding the potential risks caused by direct and indirect exposure to certain pollutants, and act as an early warning system for public health interventions [23]. Honeybees, therefore, represent a good example of the use of insects as a natural and economical monitoring system capable of detecting potentially dangerous situations for public health, and biomonitoring programs should be implemented by competent authorities following a One Health approach with an interesting return for human epidemiological studies.

### 4.2. Insects as Animal Model for Biomedical Research

During the past decade, an increasing number of insects belonging to different genera (i.e., Coleoptera, Diptera and Lepidoptera) have been used as model organisms in several life science and medical disciplines due to their worldwide distribution and environmental significance, and the conservation of their signaling pathways, energy metabolism and structural components [35,36,37]. In addition, the innate immune system of insects shares a high degree of structural and functional homology with the mammalian innate immune system [38,39]. For this reason, analysis of insect responses to pathogens can provide an indication of the vertebrate response to infection. As model hosts, insects have several advantages including low maintenance costs, the ability to obtain large quantities, their short life span and their use without major ethical constraints [40]. In fact, as invertebrates, insects are not included in animal welfare legislation and ethics guidelines. The use of insect models reinforces the importance of applying the ‘3Rs’ principles (replacement, reduction and refinement) in animal experimentation, leading to a reduction of the number of mammals and other animals in general used in research [41].

On the other end, although ethical regulations allow the use of anesthetized or immobilized live animals as a source of blood for mosquitoes, since their care and housing is expensive and time-consuming, and animal welfare has become a matter of concern, it is important to develop cheap, suitable, and effective artificial blood-feeding systems that replace live animals, taking animal welfare into appropriate consideration [42].

The fruit fly, *Drosophila melanogaster*, has been the most commonly used experimental organisms in genetic studies for more than 100 years [43]. Over the past few years, many insects have been used as in vivo alternative models for studying disease development processes, assessing microbial virulence, host resistance, and for evaluating the efficacy and toxicity of antibiotics, fungicides and other biologically active substances [44]. In particular, larvae of the greater wax moth (*Galleria mellonella*) have been widely used as experimental models to study host–pathogen interactions and the effectiveness of antimicrobial agents [40]. At present, *G. mellonella* larvae are a reliable and pertinent model for the analysis of pathogenesis and virulence factors of fungi [45].

Another application of insects in biomedical field is represented by maggot debridement therapy (MDT). This is a treatment consisting of controlled applications of cultured sterile maggot larvae to an infected chronic non-healing wound, especially in patients with impaired healing due to multi-drug resistant bacterial infection, cardiovascular or metabolic disorders [46,47,48]. The therapy consists of a three-stage process: debridement (removing the necrotic tissues by mechanical actions and by proteolytic digestion), disinfection (antimicrobial effects), and stimulation of wound healing exerted by their excretions and secretions (E/S) [49]. Two maggots, *Lucilia sericata* and *Lucilia cuprina*, are considered to be pivotal to MDT due to the antibacterial, antifungal, antiparasitic and antiviral activities of their E/S [50]. Maggot therapy is considered a modern technique in the managements of wounds and infection both in human and veterinary medicine [51].

Lastly, insects can produce a variety of antimicrobial peptides/proteins (AMPs) that have activities against bacteria, fungi, parasites, viruses and cancer cells [52]. A different number of AMPs, according to the species, can be produced in term of amino acid sequence and structures. These AMPs naturally occur during the insect life cycle and their production can be induced and/or increase during an immune response. Insect AMPs, compared with traditional antibiotics, have a unique mechanism of action and it is not easy for them to cause microbial resistance [53]. These advantages and the rich resource content of insects make AMPs excellent templates for the development of new antimicrobial drugs, for addition to food and feed as preservatives and additives. Further studies are needed to investigate structure-activity relationships, activity mechanisms, bioavailability, and synergistic effects with antibiotics.

The above-mentioned examples on different insect research lines highlight the huge plasticity insects can offer in the research field. Implementation of existing applications and establishment of new research scenarios utilizing insects should be encouraged and adopted by the public health sector.

## 5. Insect Farming

### 5.1. Insect Health

The first requirement in insect production systems is to guarantee insect health and welfare. Farmed insect can harbor a plethora of microorganisms (e.g., bacteria, viruses, fungi, protozoa and other organisms) that can be grouped in three major categories: (i) non-pathogenic (e.g., physiological microbiota); (ii) pathogenic to insects themselves; (iii) pathogenic to vertebrates, both animal and human.

These microorganisms can be introduced in reared insects by contaminated food, litter, debris, aerosol dispersion in the environment, workers or visitors, and the introduction of new-farmed specimens or other unwanted animal species. Alternatively, stress conditions or other factors can trigger covert infection already present into an overt infection with consequences for insect health and farm production. In particular, environmental conditions (e.g., high relative humidity or suboptimal temperature) or rearing conditions (e.g., high population density, non-balanced diet, and inbreeding) are the main factors that may stress insects and weaken their immune system. These factors can elicit rapid disease outbreaks resulting in reduced yield and productivity [54]. For this reason, a key goal of insect farmers, besides biosecurity measures, should be to establish and maintain a health management plan through constant monitoring of insects to identify signs of disease and act rapidly to prevent the spread of pathogens [55].

As for many other intensively reared animals, it is necessary to increase the knowledge on the susceptibility of insects to pathogens and on pathogen biology (i.e., transmission mechanisms, infection conditions) to develop guidelines for prevention and management of diseases on farm; in particular, to set up surveillance, sanitation procedures and reliable and rapid diagnostic screening protocols to minimize the risk of outbreaks and production losses.

These activities call for professionals with expertise in insect health, able to define and operate health management systems in insect farms, to control live insect markets, and avoid the spreading of pathogens. These activities will become more and more important with an increase in the number of insect farms and the intensification of a farming system in response to the increase of the feed and food market share.

In addition, the development of these farming systems requires the standardization and availability of diagnostic techniques able to detect insect pathogens. Diagnostics will be particularly useful for monitoring farm health status, and also for the certification of live insects during commercial exchanges, since the introduction of infected individuals could have serious effect on farms.

For example, among insects reared for food production, the house cricket *Acheta domesticus* is an interesting species due to its high protein content and prolificacy [56]. However, this cricket is highly susceptible to bacterial, viral and fungal pathogens, as reported in several papers [57,58]. Today the main virus affecting the reared European house cricket is the *Acheta domesticus* densovirus (AdDV), a parvovirus causing widespread morbidity and mortality in a few days in cricket farms, leading to a decline in production and even the extinction of the cricket colony. Symptoms of infection are both physical (i.e., loss of consistency, malnutrition, inhibited growth, reduced fecundity and increasing sluggishness) and behavioral (less activity) [59,60,61]. Despite the well-known effects of AdDV, there are few diagnostic protocols to detect and quantify this virus. Since AdDV is spread through oral-fecal transmission [60], the analysis of cricket frass [62] is a promising method to identify clinical symptoms and minimize disease spread without sacrificing any cricket specimens.

### 5.2. Insect Welfare

Animal welfare has been defined for farmed vertebrates in terms of the “Five Freedoms”, i.e., freedom from (1) hunger and thirst, (2) discomfort, (3) pain, injury and disease, (4) fear and distress, and (5) freedom to express natural behavior [63]. In response to this scientific claim, the European Commission has outlined the minimum requirements for animal welfare in livestock within a common legal framework through general [64], cross-cutting [65,66] and species-specific laws (limited to pigs, calves, hens and broilers). The field of application expressly excludes invertebrates. The Lisbon Treaty on the functioning of the European Union has remarked that animals are sentient beings capable of suffering [67]. It is still not clear whether the acknowledgement also applies to invertebrates. On the other hand, the EU Directive on the protection of animals used for scientific purposes includes a class of invertebrates (i.e., Cephalopods) [68]. Therefore, in the future, animal experimentation could represent a starting point for further evaluation of the welfare needs of all invertebrates, including insects. Ethical implications in the use of invertebrates in scientific research could be then extended to other aspects of our relationships with these animals, particularly concerning pest-killing procedures and intensive insect farming.

Edible insects are growing in importance from a consumer perspective in terms of novel food, and feed for farmed animals under intensive systems [69]. Therefore, identifying welfare standards and good husbandry practices is an issue of concern for veterinary public health authorities. Recent studies on pain perception [70], cognitive abilities [71], and pessimistic bias [72] have identified a variety of sophisticated responses in insects. Therefore, in his recent review, van Huis has suggested considering them “precautionary as sentient beings” [73]. On the other hand, there is limited information on practical welfare requirements for farmed insects, which might differ from vertebrates due to the considerable evolutionary distance and variability between species [69].

Although legal requirements are still lacking, pioneering insect farmers have outlined good farming practices for their niche markets, which have not been publicly available for trade secret reasons [74]. More recently, the Finnish Food Safety Authority (Evira) and the IPIFF have promoted insect welfare by applying the Five Freedoms [63] to insect farming, and have released the information open access online [75,76,77]. These guidelines suggest (a) considering species-specific physiological and ethological needs, (b) providing an adequate environment (e.g., food, water, temperature, humidity, ventilation, lighting, cleanliness, quality/quantity of substrates and enrichment materials, prevention of escapes) under mass-rearing and transport conditions, (c) preventing injuries and cannibalism (e.g., managing stocking density and providing suitable space/shelters), and d) ensuring rapid death through proper and efficient euthanasia. Different killing methods have recently been described, such as hot water, boiling vapor, freezing, and mincing [75]. Finally, insect farmers and veterinary authorities should be kept abreast of the latest science regarding the possible experiences of fear and distress in different species, and further research on this is needed [69,73].

### 5.3. Biosecurity

Insects can be farmed for a great variety of reasons ranging from sourcing valuable byproducts (e.g., bees and silkworm) to research studies, biocontrol methods, food and feed production, bio-composting and waste reduction, and recreational purpose. Farming specifications differ according to the species and intended use, but in all cases, with different level of attention, avoiding the introduction of undesired animals and/or microorganisms and contaminants or the escape of farmed species in the environment (biosecurity).

Preventing the introduction of insect pathogens and pests (e.g., other insect species, mites, spiders, birds, rodents and small mammals) into the insect farming system is crucial for animal welfare, economic productivity, food and feed safety, and public health in case of zoonotic pathogens. Biosecurity embraces all aspects of the prevention of harmful agents entering and spreading within an insect farm, or insects escaping from it. Prevention appears to be the most reliable approach to insects rearing, given the peculiarity of these animal species and of this type of farming. Therefore, the maintenance of appropriate environment and sanitary parameters, cleaning procedures, binding access procedures and quarantine as appropriate, guaranteed feed, water and rearing substrates provided, strongly contribute to sustainability and health of insect farming.

Treatments with antimicrobials and antiparasitic substances do not seem appropriate and can probably be of little use once the disease has occurred, considering the high concentration of individuals per production unit. In addition, there is no regulation about their use, and few studies have been conducted on chemical residues in farmed insects.

Currently, there are no specific biosecurity instructions for insect farming but only general guidelines on good hygiene practices for insect producers released by the International Platform of Insects for Food and Feed (IPIFF) [75]. The implementation and continuous improvement of farmed insect biosecurity programs is still a challenge for public health authorities. An efficient biosecurity system should require the implementation of dedicated management systems, including good breeding practices, good hygiene practices, good farming practices and effective pest management and pest control programs.

New, specific, complete and adapted (i.e., for insect species and infrastructures capability) biosecurity measures for this new sector must be drawn up soon by public health authorities. Insect farming requires a high level of insect health status based on daily inspections, accompanied by an appropriate set of analyses (both visual inspection and molecular test) to sustain the rearing process.

The level of biosecurity in an insect farming system should be set and achieved depending on the intended use of insects. Therefore, the highest level of biosecurity should be ensured for insects farmed for experimental infections during research activities in or to avoid interference between different pathogens that could lead to misleading results.

In the case of edible insect used for human and animal consumption, the biosecurity level should guarantee requirements to ensure food and feed safety and, therefore, consumers’ health. To date, the highest level of biosecurity can be found in approved environmentally isolated bumble bee (*Bombus* sp.) production establishments (intended for pollination) that ensure effective isolation of the production of animals from the associated facilities, and from the environment, preventing any contamination with pathogens and parasites. A lower level should be maintained in case of insects used to bio-convert vegetable waste.

In addition to the intended use for the insect, specific characteristics and the stage of development of insects should be also taken into account. For example, the level of biosecurity should be scaled up based on the risk of escape. Different containment measures should be adopted for larval and adult stages of winged insects (e.g., *Lepidoptera*) and between same stages of different orders (e.g., *Coleoptera* and *Orthopthera*).

In the context of public health, attention should be paid also to the health of farm employees to identify the potentially adverse effects of insect farming. Insects are a major source of allergens for humans, and insect asthma and allergy symptoms can be induced by bites, stings, inhalation, and ingestion [78]. Exposure to insect particles can occur in indoor and outdoor environments during daily life in non-occupational settings, as well as in occupational settings [79]. There is a large regional difference in the rate of sensitization to insect allergens, which might reflect differences in the numbers and types of insects dominant in the environment [78]. Based on the literature, daily handling of edible insects can contribute to allergies [80,81], and therefore specific behavior should be prescribed by public health authorities and adopted by insect farmers.

## 6. Future Perspectives of Insects and Public Health

The relationship between insects and public health is not new, as the role of insects as pests has been recognized since ancient times. Recently, however, it has become clear that both, negative and positive impacts of insects toward human and animal health are growing and will continue to grow in the next decades. Negative impacts of insects as pests and vectors of diseases are increasing due to climate change and globalization with geographical expansion of insect habitats and a growing possibility to host pathogens. Positive impacts are getting more consideration. The important role of insects as pollinators is widely acknowledge due to the threat of soil deterioration and biodiversity losses. Their use as food or feed is attracting great interest due to the need for sustainable protein sources. These positive impacts are changing attitudes toward them.

These quantitative and qualitative changes of insects-human relationship need to be taken into account within the public health sector. Public health is the science of protecting and improving the health of people and their communities. Protecting people from the negative effects of insects as pests and as vectors of disease is an aspect of public health. Encouraging the use of new and sustainable sources of food, protecting health and welfare of farmed insects is another aspect of public health. New responsibilities call for the update of training and education for professionals involved in these fields, namely biologists, veterinarians, doctors and others.

Public health authorities should contribute and favor the increase of innovation in mechanization, automation, processing and logistics to reduce production costs, as well as to increase the level of food and feed safety of insect mass-rearing production. They should also (a) develop feeding tables for insects and the nutritional value of substrates, conduct extensive life cycle assessments among a vast array of insect species to enable comparisons of insects with conventional feed and food sources, (b) maintain resilient genetic diversity to avoid colony collapse in insect farming systems, (c) develop voluntary best rearing practices, codes and regulatory frameworks governing insects as food and feed, as well as human health and animal welfare at the national and international levels (e.g., the Codex Alimentarius, Efsa, European Commission, FAO), and (d) improve risk assessment methodologies for risks related to mass-rearing and wild gathering in order to safeguard against the introduction of alien and invasive insect species to wild populations.

## 7. Conclusions

Threats and opportunities will arise from insects in the near future. Public health professionals should continue to address the threats, increasing their knowledge of efficient surveillance and control strategies. In addition, they should encourage the efforts of businesses in grasping the opportunity to address food security issues, develop insect safety assurance systems, and working along the whole supply chain, merging experiences from other sectors with respect to the use, control and care of insects.

## Data Availability

The data presented in this study are available within this article.
